# Multi-Omics Analysis of the Co-Expression Features of Specific Neighboring Gene Pairs Suggests an Association with Catechin Regulation in *Camellia sinensis*

**DOI:** 10.3390/genes17010117

**Published:** 2026-01-22

**Authors:** Shuaibin Lian, Feixiang Ren, Shuanghui Cai, Zhong Wang, Youchao Tu, Ke Gong, Wei Zhang

**Affiliations:** 1 College of Physics and Electronic Engineering, Xinyang Normal University, Xinyang 464000, China; 2 College of Life Sciences, Xinyang Normal University, Xinyang 464000, China

**Keywords:** neighboring gene pairs, epigenetic regulation, intergenic distance, Hi-C, transcription factors, metabolic regulation

## Abstract

Background/Objectives: The arrangement and positioning of genes on chromosomes are non-random in plant genomes. Adjacent gene pairs often exhibit similar co-expression patterns and regulatory mechanisms. However, the genomic and epigenetic features influencing such co-expression, particularly in perennial crops like tea (*Camellia sinensis*), remain largely uncharacterized. Methods: Firstly, we identified 771 specific neighboring gene pairs (SNGs) in *C. sinensis* (YK10) and investigated the contributions of intergenic distance and gene length to SNGs’ co-expression. Secondly, we integrated multi-omics data including transcriptome, ATAC-seq, Hi-C and histone modification data to explore the factors influencing their co-expression. Thirdly, we employed logistic regression models to individually assess the contributions of nine factors—ATAC-seq, H3K27ac, Hi-C, GO, distance, length, promoter, enhancer, and expression level—to the co-expression of SNGs. Finally, by integrating co-expression networks with metabolic profiles, several transcription factors potentially involved in the regulation of catechin metabolic pathways were identified. Results: Intergenic distance was significantly negatively correlated with co-expression strength, while gene length showed a positive correlation. Furthermore, these two features exerted synergistic effects with threshold characteristics and functional significance. SNGs marked by either ATAC-seq or H3K27ac peaks displayed significantly higher expression levels, suggesting that epigenetic regulation promotes co-expression. In addition, correlation analysis revealed that the expression of certain SNGs was closely associated with catechin accumulation, particularly epicatechin gallate (EGC) and epigallocatechin gallate (EGCG), highlighting their potential role in modulating tissue-specific catechin levels. Conclusions: Collectively, this study reveals a multilayered regulatory framework governing SNG co-expression and provides theoretical insights and candidate regulators for understanding metabolic regulation in tea plants.

## 1. Introduction

Mounting evidence suggests that gene organization in eukaryotic chromosomes follows non-random patterns [1]. Genes with similar expression profiles across tissues or experimental conditions often cluster within the same genomic regions [2,3], a pattern observed across numerous plant and animal species [4,5]. For instance, in the mouse genome, genes associated with immune responses and essential survival functions are frequently organized into clusters [6]. Several mechanisms have been proposed to explain the co-expression of adjacent genes, including the presence of shared promoter elements [7], and coordinated histone modifications [8]. Notably, co-expression can persist even after gene pairs become physically separated during evolution [9], likely due to their continued spatial proximity in the three-dimensional nuclear environment [10]. The higher-order folding of chromosomes facilitates the physical closeness of distant chromatin regions, enabling potential regulatory interactions among the genes located within them [11]. Recent studies have demonstrated that such spatial colocalization is functionally associated with transcriptional regulation [10,12]. Based on these findings, gene pair colocalization can be broadly classified into three types: spatially colocalized, physically neighboring, and evolutionarily neighboring [13].

The leaves of *Camellia sinensis* serve as the primary raw material for various tea beverages, which represent the most widely consumed non-alcoholic drinks globally [14]. In China, the use of tea dates back approximately 3700 years [15]. Tea leaves are rich in a variety of secondary metabolites, among which catechins—belonging to the group of tea polyphenols—are particularly prominent, typically accounting for 18% to 36% of the dry weight of tea leaves. Catechins not only contribute to the distinctive and pleasant flavor of tea but also exhibit multiple health-promoting properties, including antioxidant, anti-inflammatory, and free radical-scavenging activities [16,17]. Additionally, tea is abundant in other functional compounds such as caffeine and theanine, which impart bitterness and umami sweetness, respectively [18,19]. To date, nearly 4000 bioactive compounds have been identified in tea leaves. The astringency of tea is primarily attributed to flavonoids, while its bitterness is mainly due to caffeine [20,21].

In recent years, high-quality chromosomal assemblies have been achieved for several tea cultivars, including *C. sinensis* var. *assamica* (CSA, YK10), *C. sinensis* var. *sinensis* (CSS, Shuchazao, Biyun, Longjing 43, Tieguanyin, Huangdan), and ancient tea trees (DASZ) [22,23]. These genomic resources have facilitated the exploration of 3D chromatin architecture in tea [24]. The three-dimensional organization of chromatin is not only essential for DNA replication and chromosome recombination but also profoundly affects gene expression through modulating chromatin accessibility and regulatory element distribution, ultimately contributing to phenotypic variation [25]. Epigenetic regulation of gene expression is widespread across animals, plants, and fungi, playing crucial roles in processes such as development, disease, and environmental response [26,27]. These epigenetic modifications remodel DNA–protein interactions within chromatin, resulting in transcriptional states that are active, poised, or silenced. Consequently, they influence DNA accessibility and nuclear positioning. For instance, acetylation of histone H3 at lysine 27 (H3K27ac) is frequently associated with transcriptional activation [28]. Precise temporal and spatial regulation of transcription is vital for complex biological processes such as cell differentiation and response to environmental cues. This regulation is mediated by interactions between transcription factors (TFs) and cis-regulatory elements (CREs) [29,30]. Deciphering CREs is fundamental to understanding transcriptional networks underlying tissue specificity and phenotypic diversity. Active CRE regions often exhibit open chromatin conformations, allowing for access to regulatory proteins.

In this study, we identified 771 species-specific neighboring gene pairs in tea (*C. sinensis*), which are located adjacently in the tea genome but not in the genomes of other species. To explore the biological significance of these tea-specific gene arrangements, we integrated multiple omics-based features that potentially influence gene expression and co-expression. We addressed the following key scientific questions: (1) What are the transcriptional expression patterns of these species-specific neighboring gene pairs? Are they associated with the biosynthesis of tea flavor-related metabolites? (2) Why do specific neighboring gene pairs tend to exhibit co-expression patterns? Which factors contribute to their co-expression features? To address these questions, we focused on the *C. sinensis* cultivar Yunkang-10 (YK10) as the primary reference genome and conducted a comprehensive genomic analysis by integrating nine factors, including ATAC, H3K27ac, Hi-C, GO, distance, length, promoter, enhancer, and expression level, to investigate the co-expression features of specific neighboring gene pairs.

## 2. Materials and Methods

### 2.1. Identification of Special Neighboring Gene Pairs

**Figure 1:**
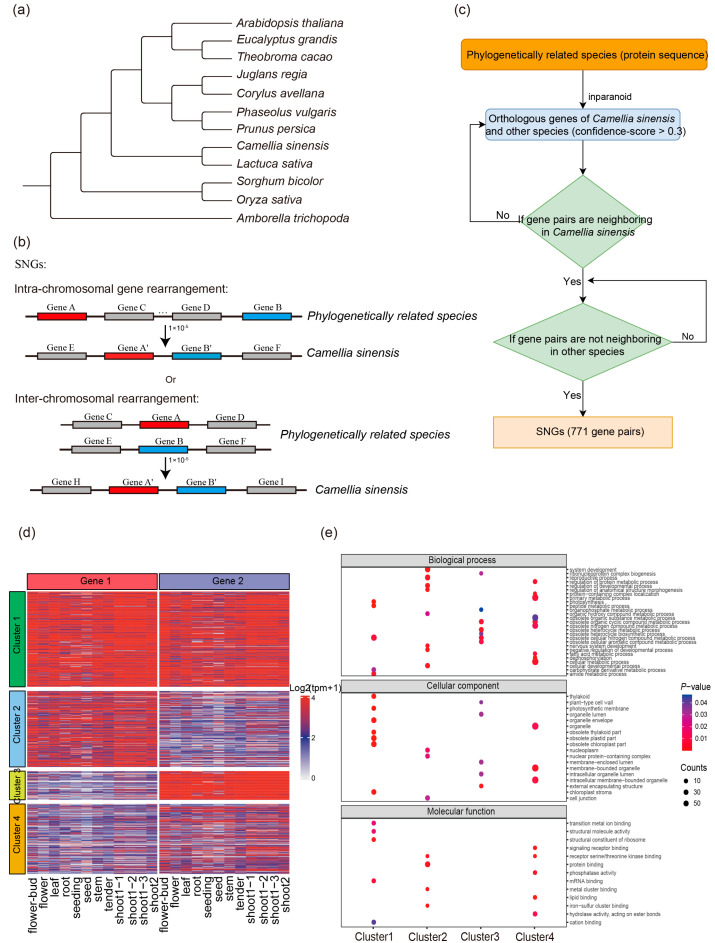
(**a**) A phylogeny of species included in this study. (**b**) Schematic representation of specific neighboring gene pairs (SNGs) (**c**) Workflow of SNG identification (**d**) Heat map of differential expression patterns of SNGs across different developmental stages and tissues (**e**) Functional enrichment analysis of each cluster.

In this study, we used the tea plant *C. sinensis* (YK10) as the focal species, along with 11 additional reference species, including eight eudicots, two monocots, and the basal angiosperm *Amborella trichopoda*. A phylogenetic tree was constructed using the Tree2GD software (version 1.0.4) based on these species [31]. The information of 11 other plant species and their phylogenetic relationships were shown in Figure 1a. We then used the InParanoid tool to identify orthologous gene pairs between tea and the other 11 species based on homology analysis [32]. The parameters for orthology inference were set as follows: E-value < 1 × 10^−5^ and confidence score ≥ 0.3, with all other parameters kept at default settings. By analyzing the locations and neighborhood relationships of orthologous gene pairs, we identified 771 special neighboring gene pairs, involving a total of 1449 genes (Table S1). In particular, neighboring gene pairs refer to gene pairs which are linear neighbors in chromosome, separated gene pairs refer to gene pairs are not linear neighbors in chromosome.

### 2.2. Quality Control and Processing of Multi-Omics Dataset

All datasets used in this study were obtained from public repositories (see Data Availability Statement for details), and their quality and reliability were assessed through multiple approaches. First, RNA-seq data were subjected to quality assessment using FastQC, followed by filtering and trimming with fastp using default parameters [33]. Cleaned reads were aligned to the YK10 reference genome using HISAT2 [34], and gene-level quantification was performed with featureCounts [35]. Gene expression levels were normalized using the TPM method. The processed RNA-seq data exhibited high mapping rates and uniform coverage, indicating accurate transcript quantification. Second, ATAC-seq and H3K27ac ChIP-seq datasets were quality-checked with FastQC and fastp. Cleaned reads were aligned to the YK10 genome using Bowtie2 [36], and low-quality alignments (Q10) were removed with SAMtools [37]. Duplicate reads were filtered using Picard, and peaks were called with MACS2 [38]. Peak reproducibility across biological replicates was evaluated using DeepTools, and peaks were successfully annotated to nearby genes using BEDTools [39]. These results confirmed the reliability of the chromatin accessibility and histone modification datasets. Third, Hi-C sequencing data were processed with FastQC and fastp, aligned to the YK10 genome, and analyzed using HiC-Pro to generate genome-wide chromatin contact matrices at resolutions of 5 kb, 10 kb, 20 kb, 40 kb, and 100 kb [40]. Matrices were normalized using the ICE method. The Hi-C contact maps exhibited well-organized interaction patterns along the main diagonal, supporting the accuracy of the chromatin interaction data. Finally, promoter regions (2 kb upstream of transcription start sites) were submitted to the PlantCARE database to identify cis-regulatory elements, and transcription factors were predicted using PlantTFDB v5.0. These analyses validated that regulatory annotations and TF predictions were of high quality [41,42].

### 2.3. Random Experiments

We used the R programming language and conducted simulations based on the complete genome of YK10. In each iteration, two genes were randomly selected and their TPM values were used to calculate the Pearson correlation coefficient. For each simulation, the number of gene pairs sampled corresponded to the number of SNGs within various distance-defined ranges. The proportion of co-expressed pairs (defined as Pearson’s r > 0.5) was recorded for each run. This process was repeated 100,000 times, and the frequency distribution of co-expression proportions across all iterations was subsequently analyzed.

### 2.4. Molecular Feature Metrics and Datasets Used

The following metrics were assessed for their potential to regulate local gene co-expression.

(a) Distance: The genomic distance between gene pairs was calculated as the absolute difference between the start site coordinates of gene1 and gene2, based on the YK10 reference annotation (gff3).
(b) Length: The average length of the transcribed region (TSS to TES) of each gene in the pairs.
(c) Hi-C: ICE-normalized Hi-C contact frequency between the 5-kb genomic bins containing the transcription start sites (TSSs) of each gene in the pair.
(d) ATAC-seq and H3K27ac ChIP-seq: These two features were included in the model as binary variables: a value of 1 indicates the presence of either an ATAC-seq or H3K27ac ChIP-seq peak in the gene; otherwise, 0.
(e) GO term sharing: The total number of shared Gene Ontology (GO) terms in the Biological Process (BP) category between the two genes in the pair. GO term matching was based on exact GO ID correspondence. GO annotations were obtained using the eggNOG-mapper database [43].
(f) Expression level difference: The relative difference in expression between the two genes in a pair.
(g) Enhancer sharing: This variable indicates whether the two genes in the pair share the same enhancer. In the model, a value of 1 is assigned if an enhancer is shared; otherwise, the value is 0.
(h) Promoter: Number of shared cis-regulatory elements in the promoter regions of SNGs.

### 2.5. Logistic Regression Models

In this study, logistic regression models were constructed using custom scripts in the R programming language (v 3.4.4), utilizing the “glm” function to assess multiple molecular features potentially associated with co-expression of specific neighboring gene pairs (SNGs). Co-expression status was encoded in a binomial format: SNGs exhibiting co-expression were labeled as positive cases (value = 1), while non-co-expression SNGs were treated as negative cases (value = 0). To ensure class balance in the dataset, an equal number of negative samples were randomly selected from the non-co-expressed group to match the number of positive cases.

Each model was trained on 80% of the gene pairs, randomly sampled without replacement, maintaining an equal proportion of positive and negative examples. The remaining 20% of gene pairs were used as the test set. Model performance was evaluated by calculating the area under the receiver operating characteristic curve (AUC), which reflects the ability of the trained model to correctly classify the co-expression status. This entire sampling and modeling process was repeated 50 times, and the mean AUC across all iterations was reported as a robust measure of predictive accuracy.

### 2.6. Construction of Weighted Gene Co-Expression Network

Weighted gene co-expression network analysis (WGCNA) was conducted using the WGCNA R package (version 4.3.2) to explore the potential associations between the expression profiles of specific neighboring genes (SNGs) and metabolite accumulation. A total of 1449 SNGs and 6 metabolites were included to construct a signed co-expression network based on Pearson correlation coefficients. The soft-thresholding power for network construction was set to 14, the minimum module size was defined as 30, and the threshold for module merging was set at 0.25; all other parameters were maintained at their default settings. The resulting module networks were visualized using Cytoscape software (version 3.9.0).

### 2.7. Statistical Methods

Statistical significance between two independent samples was assessed using the Mann–Whitney U test (function ‘wilcox.test’ in software R version 4.3.2). The default significance threshold was set at 0.05. As a non-parametric test, the Mann–Whitney U method is designed to compare the medians of two independent groups and does not assume a normal distribution of the data, making it particularly suitable for skewed or non-normally distributed datasets.

To robustly estimate the error bars of proportions presented in bar plots, we employed a bootstrap resampling approach. Specifically, for each group of binary observations, we performed 1000 iterations of resampling with replacement, each time generating a bootstrap sample of the same size as the original dataset. For each resampled dataset, the proportion of positive cases was calculated, and the standard deviation of these 1000 proportions was taken as the standard error. This standard error was subsequently used to construct the error bars in the plots.

## 3. Results

### 3.1. Identification and Expression Patterns of Specific Neighboring Gene Pairs (SNGs)

First, based on the phylogenetic relationships with *C. sinensis*, we selected 11 representative plant species to construct a phylogenetic tree (Figure 1a) (see Section 2 for details). In this study, we identified a specific category of gene pairs with unusual genomic positioning—those that are not adjacent in other species but appear as neighboring genes in the tea genome (Figure 1b). We define such gene pairs as specific neighboring gene pairs (SNGs), referring to genes which were separated in the evolutionary past but are now neighbors. To identify these SNGs, we first determined the sets of orthologous gene families between *C. sinensis* and each of the 11 selected species. For each orthologous group, we first examined whether the gene pair from *C. sinensis* is located adjacent to one another on the genome. If the two tea genes are adjacent, we then assessed whether their corresponding orthologs in the other species are also neighbors. If not, the pair is classified as an SNG. Subsequently, we intersected the SNGs identified across all 11 species and ultimately obtained a total of 771 SNGs. These represent gene pairs that are consistently adjacent only in *C. sinensis* but not in any of the other 11 species (Figure 1b,c) (see Section 2 for details). To investigate the expression patterns and functional characteristics of the 771 identified SNGs during natural growth and development, we first performed expression clustering analysis. Based on their transcriptional profiles, SNGs were grouped into four distinct clusters (Figure 1d).

Notably, a substantial fraction of SNGs (272 pairs) exhibited coordinated high expression within the same cluster, indicating potential co-regulation. To further explore the biological roles of SNGs, we conducted Gene Ontology (GO) enrichment analysis for each cluster. Genes in cluster 1 showed upregulation of various metabolism-related processes, particularly enriched in amide metabolic process, peptide metabolic process, and carbohydrate derivative metabolic process. These genes were also significantly associated with photosynthesis, a core process of carbon metabolism.

Functional annotations indicated that most genes in cluster 1 encode proteins involved in nitrogen compound metabolism and photosynthetic activity, with additional enrichment for ion binding and mRNA binding, suggesting dual roles in basic metabolism and post-transcriptional regulation. Cluster 2 was enriched in genes involved in the organic hydroxy compound metabolic process and those related to metal cluster binding. These genes are potentially involved in redox-related metabolic pathways and play critical roles in cell development and differentiation, reflecting the plant’s strategy of coordinating growth and stress responses through metabolic reprogramming. Genes in cluster 3 were significantly enriched in secondary metabolism-related pathways, including heterocycle metabolic process, aromatic compound metabolic process, and organophosphate metabolic process. This pattern suggests that genes in this module are likely involved in defense responses and the biosynthesis of quality-related secondary metabolites. In contrast, cluster 4 exhibited the most diverse metabolic annotation. Genes in this group were involved in a wide range of pathways, including cellular metabolic process, primary metabolic process, nitrogen compound metabolic process, and fatty acid metabolic process. This cluster also contained a substantial number of genes encoding catalytic proteins such as hydrolases acting on ester bonds and phosphatases, indicating a central role in macromolecule degradation and membrane-bound organelle function.

Taken together, the four clusters displayed a trend of functional complementarity in metabolic regulation. Specifically, cluster 4 may represent a key regulatory module for primary metabolism, while cluster 3 is more likely to be involved in the regulation of specialized secondary metabolism. This functional divergence underscores the complexity and diversity of metabolic strategies employed by tea plants in response to environmental cues and further highlights the role of SNG expression patterns in enriching gene functional diversity.

### 3.2. Genomic Distance Is Negatively Correlated with SNGs’ Co-Expression

**Figure 2:**
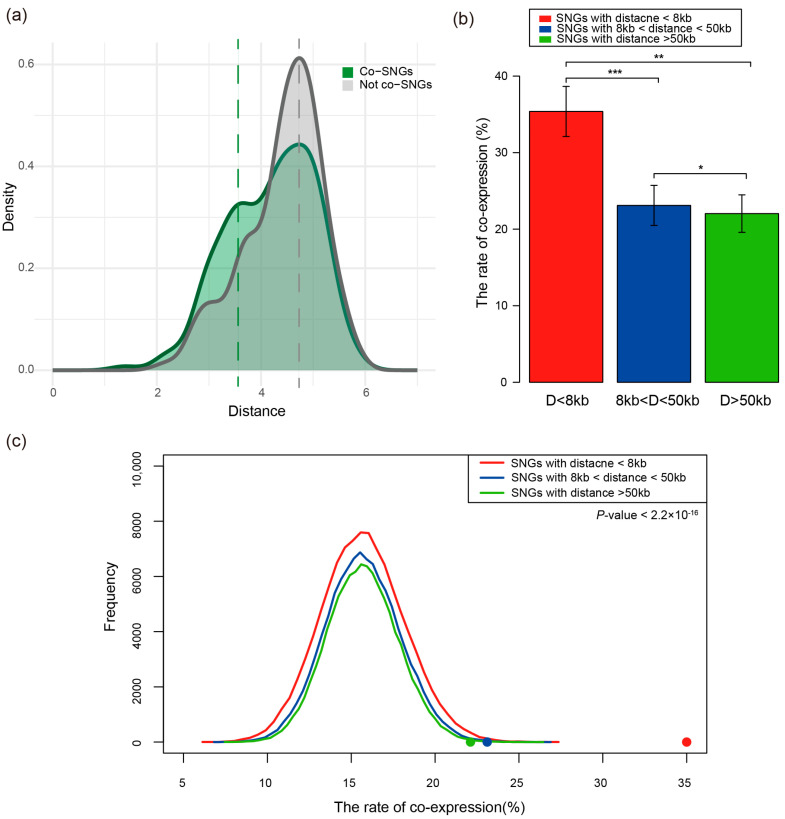
(**a**) Distribution of the absolute distance between Co-SNGs and non-co-SNGs. (**b**) Co-expression ratios of SNGs in three defined genomic distance ranges. (**c**) The frequency distributions of co-expression ratios from 10,000 randomized experiments across three distance ranges. Curves of different colors represent the distributions for different genomic distance groups, with each randomization sampling the same number of gene pairs as in the real data. Error bars were calculated by bootstrapping. Significance values calculated from the Mann–Whitney U test are shown (* *p* < 0.05, ** *p* < 0.01, *** *p* < 0.001).

We first calculated the Pearson correlation coefficient and intergenic distance for each pair of specific neighbor genes (SNGs). The analysis indicated a slight negative trend between the Pearson correlation coefficients of SNGs and their intergenic distances; specifically, Pearson correlations tended to decrease slightly as intergenic distances increased (Figure S1a). Next, gene pairs with Pearson correlation coefficients greater than 0.5 were defined as co-expressed SNGs (co-SNGs), while the remaining pairs were classified as not co-expressed SNGs (not co-SNGs). Comparing the distance distributions of these two groups showed that co-SNGs have shorter intergenic distances than not co-SNGs (Figure 2a), suggesting that shorter distance may promote co-expression.

To further investigate the effect of distance, all SNGs were divided into three groups based on intergenic distance: short-distance group (distance < 8 kb, n = 212), medium-distance group (distance between 8 kb and 50 kb, n = 264), and long-distance group (distance > 50 kb, n = 295). The co-expression proportions were highest in the short-distance group (35.38%), followed by the medium-distance group (23.11%), and lowest in the long-distance group (22.03%) (Figure 2b). The co-expression proportion in the short-distance group was approximately 53.09% and 60.50% higher than that in the medium- and long-distance groups, respectively, with the differences being statistically significant (Mann–Whitney U test, *p* < 0.01). This indicates that shorter intergenic distances significantly strengthen co-expression levels. To ensure the observed phenomenon was not due to chance, we conducted randomization experiments by randomly selecting the same number of gene pairs as the SNGs and calculating their co-expression proportions, repeating the sampling 10,000 times. The results showed that the co-expression proportion of real SNGs was significantly higher than that of the randomized groups (Mann–Whitney U test, *p* < 2.2 × 10^−16^, Figure 2c), further validating the negative correlation between distance and co-expression.

In summary, our results clearly demonstrate that physical distance between special neighbor genes is significantly negatively correlated with their co-expression level: the shorter the distance, the higher the likelihood of co-expression.

### 3.3. Gene Length Is Positively Correlated with SNGs’ Co-Expression

To further explore the factors influencing the co-expression of SNGs, we evaluated whether the average gene length of each SNG pair is associated with their expression correlation. Specifically, we calculated the Pearson correlation coefficient between the expression levels of each gene pair and examined its relationship with their average gene length. The analysis revealed a positive correlation between average gene length and Pearson coefficient, indicating that SNGs with longer average lengths tend to exhibit stronger expression correlation (Figure S1b).

**Figure 3:**
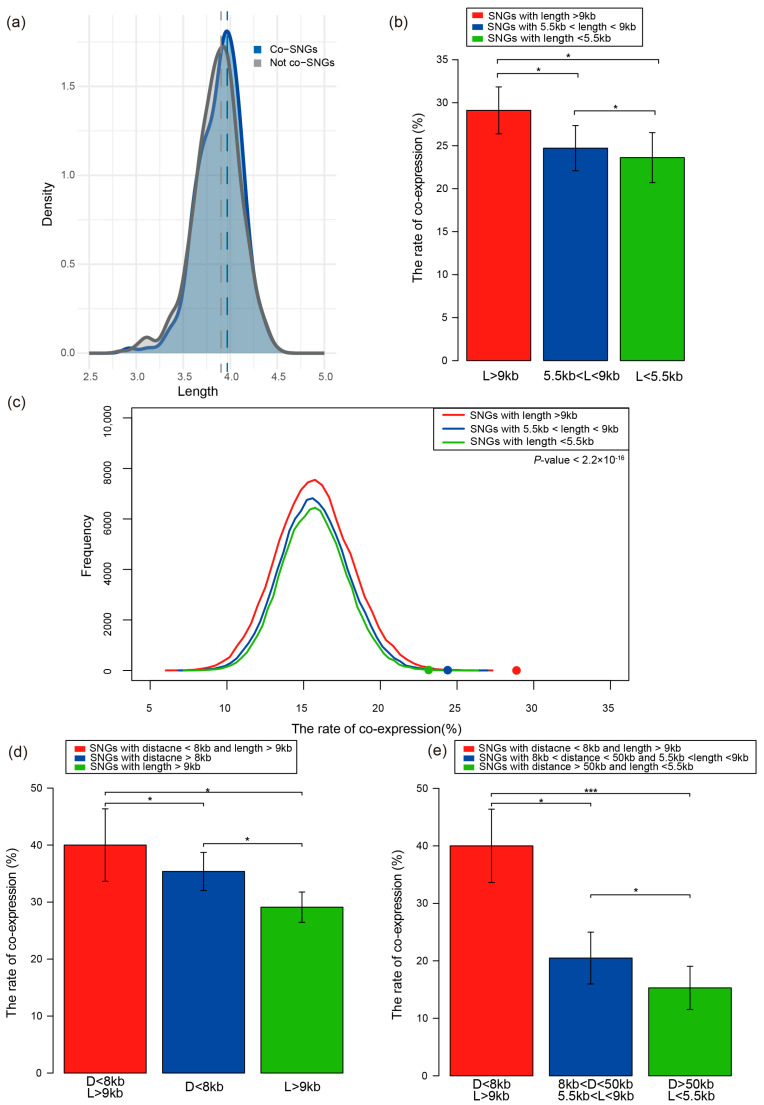
(**a**) Distribution of the average length between Co-SNGs and non-co-SNGs. (**b**) Co-expression ratios of SNGs in three defined average gene length ranges. (**c**) The frequency distributions of co-expression ratios from 10,000 randomized experiments across three length ranges. (**d**) Co-expression ratio under the shortest distance and longest gene length condition. (**e**) Co-expression ratios under combinations of genomic distance and gene length. Error bars were calculated by bootstrapping. Significance values calculated from the Mann–Whitney U test are shown (* *p* < 0.05, *** *p* < 0.001).

We subsequently observed that co-expression SNGs (co-SNGs) tended to have longer average gene lengths, with their density distribution curve clearly shifted toward longer lengths compared to non-co-expression SNGs (not co-SNGs) (Figure 3a). To further investigate the influence of average gene length on SNG co-expression, all SNGs were grouped into three categories based on their average gene length: short-length group (length < 5.5 kb, n = 216), medium-length group (length between 5.5 kb and 9 kb, n = 263), and long-length group (length > 9 kb, n = 292). The analysis revealed a gradual increase in the co-expression ratio with increasing gene length. The long-length group exhibited a co-expression ratio of 29.11%, significantly higher than that of the medium-length group (24.71%) and the short-length group (23.61%) Specifically, the co-expression ratio in the long-length group increased by approximately 17.81% and 23.30% compared to the medium- and short-length groups, respectively. These differences were statistically significant (Mann–Whitney U test, *p* < 0.05, Figure 3b), further supporting the notion that longer average gene length promotes co-expression. In addition, to determine whether this observation was due to random chance, we performed a randomization test. We randomly sampled the same number of gene pairs and calculated the co-expression ratio, repeating this process 10,000 times. The results showed that the frequency of co-expression ratios in the randomized groups was significantly lower than that observed in the actual SNGs (Mann–Whitney U test, *p* < 2.2 × 10^−16^, Figure 3c).

### 3.4. Gene Length and Genomic Distance Synergistically Strengthen SNGs’ Co-Expression

We also investigated whether the combination of intergenic distance and average gene length could synergistically strengthen the co-expression level of SNGs. We first selected SNGs that simultaneously met the criteria of distance < 8 kb and average length > 9 kb. The results showed that the co-expression ratio under this combined condition reached 40.00%, which was significantly higher than that of SNGs with only short distance (distance < 8 kb, 35.38%) or only long length (length > 9 kb, 29.11%), corresponding to increases of 13.14% and 37.41%, respectively. This suggests a stronger synergistic effect of the combined factors, and that distance may have a greater impact on co-expression than length, with an enhancement of 21.54% (Mann–Whitney U test, *p* < 0.05, Figure 3d). To further analyze the effect of different combinations of distance and length on SNG co-expression, we evaluated two additional combination groups. Besides the “short distance and long length” group which exhibited the highest co-expression, the other two groups—short distance and short length (distance < 8 kb & length < 5.5 kb) and medium distance and medium length (distance with between 8 kb and 50 kb & length with between 5.5 kb and 9 kb)—showed markedly lower co-expression ratios of 20.48% and 15.29%, respectively. These values were more than halved compared to the most optimal group (Mann–Whitney U test, *p* < 0.05, Figure 3e). We further compared the combinatorial effects with those of the corresponding individual factors. In the medium distance and medium length group, the co-expression ratio dropped to 20.48%, while the ratios for medium distance and medium length alone were 23.11% and 24.71%, respectively, representing reductions of 11.38% and 17.12%. Similarly, in the group with long distance (distance > 50 kb) and short length (length < 5.5 kb), the co-expression ratio was only 15.29%, significantly lower than that of long distance (22.03%) or short length (23.61%) alone, showing decreases of 31.82% and 35.24%, respectively (Figure S1c,d).

In summary, our analysis demonstrates that average gene length contributes to the enhancement of SNG co-expression, and that short intergenic distance combined with long gene length exhibits a synergistic effect. However, once either factor exceeds its effective range, the combined effect may diminish or even suppress co-expression. These findings highlight a complex interplay between intergenic distance and gene length in the regulation of SNG co-expression.

### 3.5. ATAC-Seq and H3K27ac Peaks Strengthen the Transcriptional Expression of SNGs

**Figure 4:**
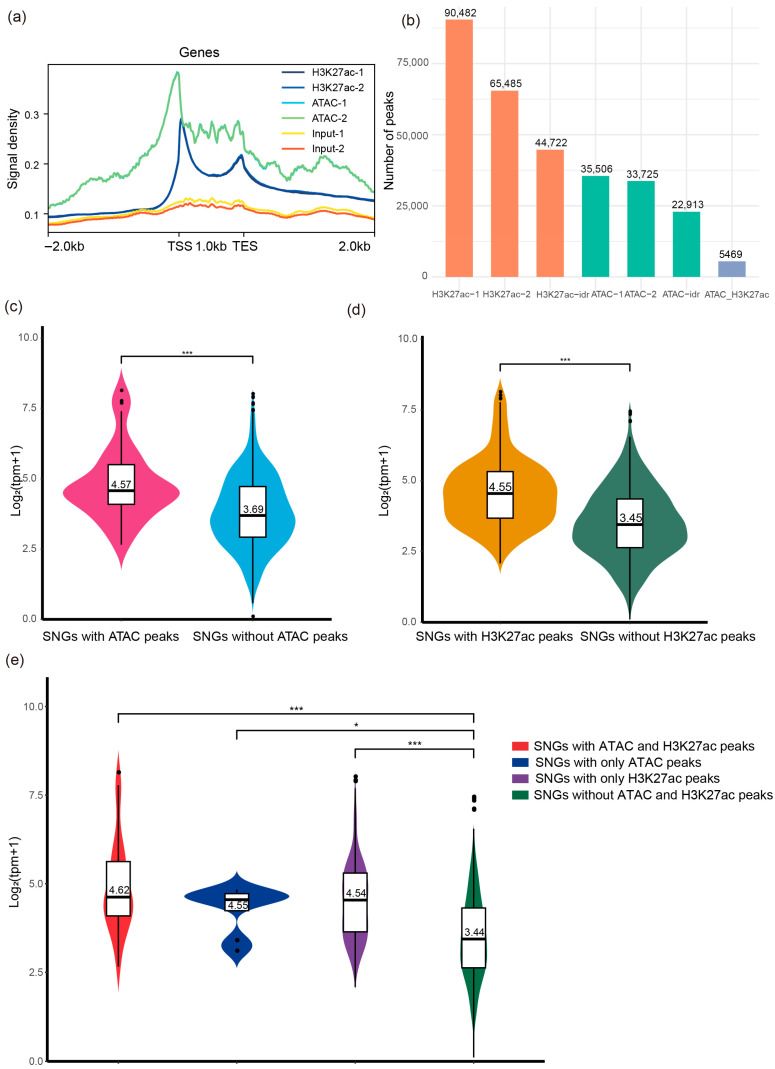
(**a**) Read depth in the 2 kb upstream and downstream of the transcription start sites (TSSs) and transcription end sites (TESs) of genes. (**b**) Number of ATAC-seq and H3K27ac peaks. (**c**) Expression levels of SNGs annotated with ATAC-seq peaks. (**d**) Expression levels of SNGs annotated with H3K27ac peaks. (**e**) Expression levels of SNGs annotated with H3K27ac peaks and ATAC-seq peaks. Significance values calculated from the Mann–Whitney U test are shown (* *p* < 0.05, *** *p* < 0.001).

Given that non-coding intergenic regions are enriched in various transcriptional regulatory elements, a genome-wide analysis of these elements is essential for understanding their influence on transcriptional activity [44]. To this end, we processed publicly available raw datasets of ATAC-seq and H3K27ac ChIP-seq from recent studies (see Section 2 for details). Our analysis revealed a strong enrichment of both ATAC-seq and H3K27ac ChIP-seq signals around transcription start sites (TSSs) (Figure 4a), suggesting a potential role in transcriptional activation. We then identified peaks from both datasets using the MACS2 algorithm and retained those consistently detected in biological replicates (Figure 4b). Since ATAC-seq peaks, which represent accessible chromatin regions (ACRs), often encompass promoters and enhancers. We further overlapped H3K27ac peaks located in intergenic regions with ATAC-seq peaks. Regions showing co-occurrence were defined as active enhancers (n = 1706) (Figure S2, Table S2).

To investigate whether the presence of ATAC-seq or H3K27ac peaks contributes to elevated transcriptional expression of SNGs, we annotated SNGs with both ATAC-seq and H3K27ac signals (see Section 2 for details). We then compared expression levels between SNGs with and without peak annotations. The results showed that SNGs annotated with either ATAC-seq or H3K27ac peaks exhibited significantly higher expression, with median values of 4.57 and 4.55, respectively, compared to 3.69 and 3.45 in the non-annotated groups. This corresponds to an expression increase of 23.85% and 31.89%, respectively (Mann–Whitney U test, *p* < 0.001; Figure 4c,d). We further examined whether co-occurrence of both ATAC-seq and H3K27ac peaks exerts a synergistic effect on gene expression. SNGs carrying both peaks displayed the highest expression levels, with a median of 4.62, representing a 34.30% increase compared to the non-annotated group. To exclude potential confounding effects between the two peak types, we analyzed SNGs annotated with either ATAC-seq or H3K27ac peaks alone. Both subsets showed significantly higher expression than the non-annotated group, with no significant difference between them, indicating comparable regulatory strength. (Mann–Whitney U test, *p* < 0.001, Figure 4e). Collectively, these results demonstrate that annotation with ATAC-seq and/or H3K27ac peaks is associated with elevated transcriptional expression of SNGs.

### 3.6. Logistic Regression Model Indicates the Molecular Features of SNGs’ Co-Expression

To systematically evaluate the influence of multiple molecular features on SNG co-expression, we trained a logistic regression model by integrating nine factors, including ATAC, H3K27ac, Hi-C, GO, distance, length, promoter, enhancer, and expression level, to investigate the co-expression features of specific neighboring gene pairs. We used 80% of the SNG pairs for training and evaluated its performance on the remaining 20%.

**Figure 5:**
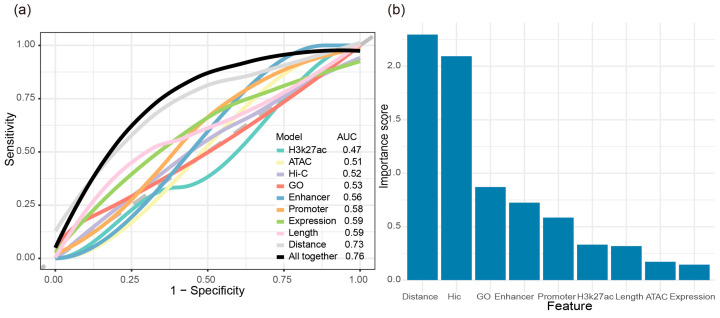
(**a**) Receiver operating characteristic (ROC) curve of predicting Co-expression for several molecular features (logistic regression; N = 771 for Co-SNGs and non-Co-SNGs; see Section 2 for details). (**b**) Relative importance of each molecular feature in predicting co-expression.

Firstly, we evaluated the relationships between these features and SNG expression correlation (measured by Pearson correlation coefficient), and further assessed their discriminative power in a logistic regression model (Figure 5a). Preliminary analyses showed that most features exhibited weak associations with expression correlation, and pairwise correlations between features were generally insignificant. Notably, only the Hi-C spatial interaction frequency showed a stable negative correlation with intergenic distance (r = −0.286), indicating that gene pairs with shorter distances tend to have higher spatial interaction frequencies (Figure S3). Given that spatial proximity may influence co-expression, and that gene pairs with closer TSSs usually have stronger spatial interactions, we further examined how varying Hi-C strength affects SNG co-expression. The results showed a negative correlation between Hi-C interaction frequency and co-expression levels among SNGs (r = −0.453). Specifically, co-expression decreased when Hi-C strength was below 10, but significantly increased when the strength exceeded 10 (Figure S4a). Combined with the negative correlation between Hi-C and intergenic distance, this pattern likely reflects that SNGs with high Hi-C strength tend to have shorter intergenic distances, indirectly enhancing their co-expression. In the logistic regression model, however, the average AUC for the Hi-C feature was only 0.52, indicating limited discriminative power.

To further explore the connection between spatial regulation and co-expression, we examined whether SNGs share active enhancers identified by overlapping ATAC-seq and H3K27ac ChIP-seq peaks (see Section 2 for details). A total of 43 SNG pairs were annotated as sharing active enhancers, but their co-expression levels were not higher than those of other SNGs (Figure S4b), and their average AUC in logistic regression was also modest (0.52; Figure 5a). In terms of functional annotation, we quantified the number of shared promoter elements and shared GO biological process (BP) terms among SNGs. We found 763 SNG pairs sharing promoter elements and 219 sharing BP terms. Both features showed a strong positive correlation with co-expression proportion, with Pearson r values of 0.546 and 0.766, respectively (Figure S4c,d). Their corresponding average AUCs in logistic regression were 0.58 and 0.53, suggesting moderate predictive power (Figure 5a).

Next, we further assessed whether ATAC-seq and H3K27ac annotations also contribute to SNG co-expression. Despite their known role in boosting gene expression, the presence of either ATAC-seq or H3K27ac peaks did not strengthen co-expression levels (Figure S4b). In logistic regression models, the average AUCs for SNGs annotated with ATAC-seq or H3K27ac peaks alone were only 0.47 and 0.51, respectively, indicating weak predictive power (Figure 5a). We also examined whether expression divergence between SNGs correlates with co-expression levels. By standardizing expression differences across tissues were calculated as the absolute difference between their average expression levels, divided by the mean expression level of the pair. we divided SNGs into two groups based on whether their expression difference was greater than or less than 1. SNGs with smaller expression differences (expression < 1) showed slightly higher co-expression levels, and their average AUC in the logistic regression model reached 0.59, outperforming most single features (Figure 5a).

Finally, among all individual features, intergenic distance showed the highest average AUC in the logistic regression model (AUC = 0.73), making it the most effective predictor of SNG co-expression. When all features were combined in a single integrated model, the resulting AUC exceeded that of any individual feature, highlighting the complementary roles of different molecular characteristics in explaining local gene co-expression (Figure 5a). Moreover, logistic regression analysis further confirmed that gene distance is positively associated with co-expression, whereas gene length shows a negative association, consistent with our previous statistical observations (Figure S5a).

To further evaluate the relative importance of various molecular features in predicting the co-expression of SNGs, we used the “varImp” function from the R package “caret” to assess each variable’s contribution within the logistic regression model. The results showed that genomic distance remained the most informative feature, exhibiting the highest variable importance score. Although Hi-C alone yielded a relatively low AUC when modeled individually, its importance ranked second in the integrated model, suggesting that Hi-C-mediated spatial interactions still play a role in regulating SNG co-expression (Figure 5b).

### 3.7. Interaction Effects Between Genomic Distance and Other Molecular Features on SNG Co-Expression

Building on the Logistic regression model, we further investigated whether genomic distance interacts with other molecular features to influence SNGs’ co-expression. If such interactions exist, it would suggest that the effect of molecular features on co-expression is modulated by the distance between gene pairs, thereby revealing a more nuanced regulatory mechanism.

**Figure 6:**
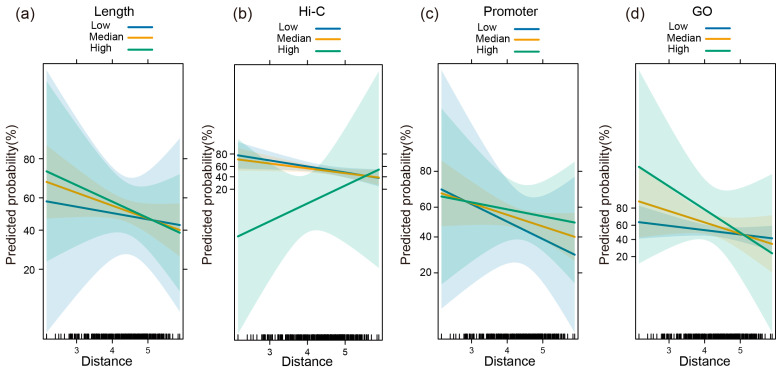
(**a**) Interaction effects between genomic distance and length on prediction frequency. (**b**) Interaction effects between genomic distance and Hi-C on prediction frequency. (**c**) Interaction effects between genomic distance and promoter on prediction frequency. (**d**) Interaction effects between genomic distance and GO on prediction frequency.

First, we incorporated interaction terms between distance and other molecular features into the logistic regression model to investigate how their combined effects influence the probability of SNG co-expression. The interaction effects were visualized using the “effects” package in R. In the interaction between distance and gene length, we observed that at shorter distances, SNGs with greater gene length exhibited the highest predicted co-expression probability (76.77%), which gradually decreased as the distance increased—consistent with previous observations (Figure 6a). In the interaction between distance and Hi-C contact frequency, the highest co-expression probability (78.19%) was associated with low Hi-C intensity at shorter distances. As distance increased, the predicted probabilities for low and medium Hi-C frequencies decreased, while those for high Hi-C frequency increased, reaching 53.90% at the longest distances (Figure 6b). These results suggest that although Hi-C interactions may negatively correlate with co-expression at short distances, they may exert a positive regulatory effect when the distance between SNGs exceeds a certain threshold.

A similar pattern was observed in the interaction between distance and shared promoter elements. At very short distances, SNGs sharing the fewest promoter elements had the highest predicted co-expression probability (72.74%) (Figure 6c). While overall co-expression probability declined with increasing distance, SNGs sharing the greatest number of promoter elements eventually surpassed the other groups at certain distance ranges. This indicates that excessive promoter sharing between closely located genes may compromise their co-expression coordination. Finally, in the interaction between distance and shared Gene Ontology biological process (GO BP) terms, SNGs located in close proximity and annotated with the same GO BP term exhibited the highest predicted co-expression probability (up to 97.89%), which gradually declined with increasing distance (Figure 6d). Similarly, in the interaction between expression differences and distance, SNGs with minimal expression differences exhibited the highest predicted co-expression frequency at short distances, which gradually declined as the distance increased. Notably, at longer distances, SNGs marked with H3K27ac peaks showed higher predicted co-expression frequencies compared to those without H3K27ac annotation. In contrast, the interaction effects between distance and either enhancer or ATAC-seq peaks were not pronounced (Figure S5b). Collectively, these findings highlight the complexity and diversity of regulatory mechanisms underlying SNG co-expression and demonstrate that changes in genomic distance can significantly modulate the regulatory influence of other molecular features.

### 3.8. Co-Expression Network Analysis

**Figure 7:**
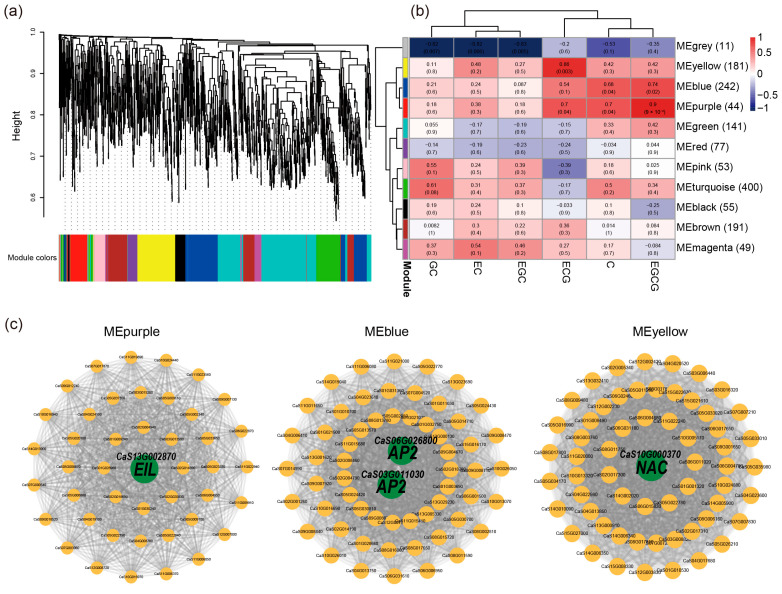
(**a**) Hierarchical cluster tree showing the 11 modules obtained by weighted gene coexpression network analysis (WGCNA). The gray modules represent genes that were not assigned to specific modules. Each branch in the tree points to a gene. (**b**) Matrix of module-metabolite associations. The data of gene expression profiles for the SNGs and the metabolite accumulation were combined to perform the WGCNA. Correlation coefficients and *p*-values between modules and metabolites are shown at the row-column intersections, with the numbers in parentheses indicating the number of genes contained in each module. (**c**) Network visualization of hub genes in four modules, highlighting transcription factors (TFs) potentially involved in metabolite regulation.

Weighted Gene Co-expression Network Analysis (WGCNA) is a highly robust method for classifying genes via Hierarchical clustering of gene co-expression networks. To explore the metabolic significance of gene modules, we correlated gene expression profiles with the accumulation levels of catechins (Figure 7a) (see Section 2 for details). A total of 1449 specific neighboring gene pairs were included in the WGCNA analysis. After merging similar modules, eleven modules were identified, each comprising between 44 and 400 genes. Among these, three modules showed significant correlations with metabolites (correlation coefficient r ≥ 0.7, *p* < 0.05). Specifically, the MEyellow module contained 181 genes, accounting for 12.5% of all analyzed genes, and was significantly associated with epicatechin gallate (ECG). The MEblue and MEpurple modules contained 242 (16.7%) and 44 (3.0%) genes, respectively, and were both significantly associated with epigallocatechin gallate (EGCG). The MEyellow and MEpurple modules were highly correlated with each other (r > 0.85), indicating that their gene networks may share similar regulatory features involved in catechin biosynthesis (Figure 7b).

Hub genes within modules are typically regarded as representative of the module’s biological function. Therefore, we constructed co-expression networks based on the top 60 genes with the highest module membership (key module eigengene-based connectivity, KME) values from these five modules, selecting transcription factors (TFs) as key hub genes (Figure 7c). In the purple module, a EIL TF was identified, which may play a critical role in ECG. In the blue and yellow modules, tow TFs belonging to AP2 and NAC families were identified, potentially involved in EGCG. Collectively, these results suggest that these transcription factors may contribute to the regulation of metabolite accumulation across different tea plant cultivars.

### 3.9. SNGs Associated with the Content of Catechins, Especially EGC and EGCG

**Figure 8:**
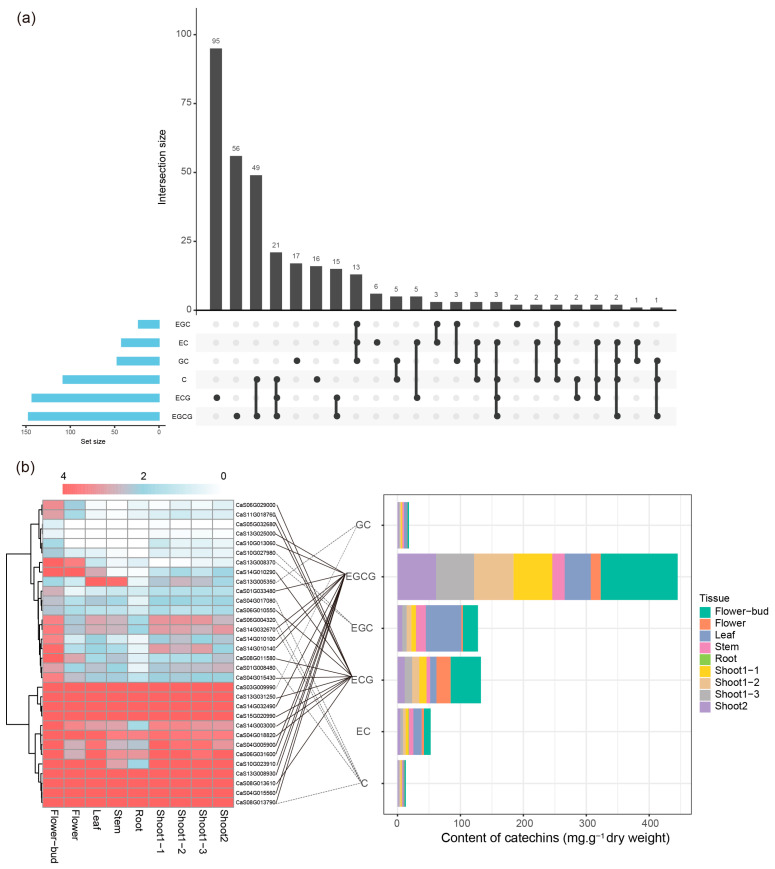
(**a**) Upset plot showing the results of correlation analysis between SNG expression and catechin accumulation (correlation ≥ 0.5). (**b**) Tissue accumulation profiles of catechins corresponding to SNG genes with strong correlation (r ≥ 0.9) with catechin levels. Expression data are plotted as log10 values, and solid lines indicate genes associated with ECG and EGCG accumulation. GC, gallocatechin; EGCG, epigallocatechin gallate; ECG, epicatechin gallate; EGC, epigallocatechin; EC, epicatechin; C, catechin.

Following this, we performed transcriptome and metabolite correlation analysis for tea SNGs. Among 1449 SNG genes, 324 genes exhibited significant correlations between their tissue-specific expression patterns and catechin accumulation (correlation coefficient r ≥ 0.5, Pearson’s correlation test, *p* < 0.05), including gallocatechin (GC), epigallocatechin gallate (EGCG), epicatechin gallate (ECG), epigallocatechin (EGC), epicatechin (EC), and catechin (C). Notably, 151 of these 324 correlated genes accounted for 46.6% and were associated with the accumulation of ECG and EGCG (Figure 8a). Further analysis revealed that 32 genes showed strong correlations with catechin tissue accumulation (r ≥ 0.9, Pearson’s correlation test, *p* < 0.05), among which 12 and 13 genes were highly correlated with EGC and EGCG, respectively (Figure 8b). Collectively, these results suggest that SNG expression is closely associated with catechin accumulation, especially EGC and EGCG, and could play an important role in regulating tissue-specific catechin levels and tea quality.

## 4. Discussion

Previous studies have shown that the linear arrangement of genes in plant genomes is not random, and early research in *Arabidopsis thaliana* revealed that physically adjacent genes are more likely to exhibit coordinated expression compared with randomly distributed gene pairs [45]. Subsequently, several studies have investigated the regulation of gene expression from the perspective of chromatin, including chromatin accessibility and histone modifications, providing new insights into gene regulatory mechanisms. For example, ATAC-seq analyses in bread wheat indicated a strong association between gene transcriptional activity and chromatin accessibility [46], and in the tea plant (*C. sinensis*, TGY) genome, chromatin accessibility and H3K27ac histone modifications were also found to be highly correlated with gene expression [24]. Therefore, we identified 771 species-specific neighboring gene pairs in tea, and integrated multiple omics datasets to explore their co-expression patterns and potential regulatory factors.

In addition, a recent study performed transcriptomic and metabolomic analyses of young shoots of seven tea cultivars and inferred candidate transcription factors (TFs) associated with characteristic metabolites based on WGCNA [47]. In this study, we similarly constructed co-expression networks based on SNGs and identified three gene modules significantly associated with catechin metabolism. Key transcription factors were detected within these modules, including EIL (CaS13G002870), AP2 (CaS06G026800 and CaS03G011030), NAC (CaS10G000370). EIL transcription factors act as central regulators of the ethylene signaling pathway, playing crucial roles in plant development, hormonal crosstalk, and stress responses [48]. AP2 transcription factors have been reported to participate in different steps of catechin biosynthesis [49], while NAC has been identified as a key regulatory hub in the biosynthesis pathways of theanine, catechins, and caffeine [50]. Considering the significant enrichment of these transcription factors in catechin-related modules and their strong correlations with module eigengenes, we speculate that they may cooperatively regulate catechin metabolism in tea plants.

Despite uncovering multilayered regulatory mechanisms of SNG expression and co-expression, this study has limitations. Firstly, we have yet to precisely determine the critical threshold of intergenic distance at which its enhancing effect on co-expression begins to diminish, and to fully elucidate how other factors intervene in this process. Secondly, the biological basis and evolutionary significance underlying the formation of SNGs—why certain genes emerge adjacent and exhibit specific expression patterns following speciation or gene duplication—require further investigation. Thirdly, previous studies have shown that metabolite accumulation and regulation are highly cell-type- and subcellular-specific [51]. Considering that our co-expression analysis is based on bulk RNA-seq data from different tea tissues, future integration of single-cell or spatial transcriptomic data and subcellular localization experiments will help elucidate the cell-type-specific regulation of SNGs and their roles in metabolic pathways.

## 5. Conclusions

In this study, we systematically identified specific neighboring gene pairs (SNGs) in tea plant and comprehensively analyzed their expression patterns and the regulatory factors underlying their co-expression. Initially, we performed clustering of SNGs based on expression levels and found that genes within distinct expression clusters were enriched in metabolism-related biological processes, indicating that SNGs play important roles in metabolic regulation in tea plants.

Furthermore, we revealed the influence of intergenic distance and average gene length on SNG co-expression patterns: (1) intergenic distance exhibited a significant negative correlation with co-expression strength, where shorter distances corresponded to stronger co-expression, and co-expression decreased markedly as distance increased; (2) average gene length was positively correlated with co-expression, with longer gene pairs generally exhibiting higher co-expression levels, while shorter lengths were associated with reduced co-expression. Based on these observations, we explored the combined effect of distance and length, discovering that they synergistically strengthen SNG co-expression within certain thresholds, but this enhancement weakens or disappears once distance or length surpass critical values, suggesting complex threshold effects and nonlinear relationships in co-expression regulation.

In addition, chromatin accessibility and H3K27ac histone modifications significantly promote SNG transcriptional expression. We observed that SNGs annotated with both ATAC-seq and H3K27ac peaks displayed markedly elevated transcription levels, with a stronger combined effect, implying a coordinated role of these epigenetic marks in regulating adjacent gene expression. Logistic regression modeling incorporating multiple molecular features demonstrated that (1) intergenic distance is the most influential factor affecting SNG co-expression; (2) SNG co-expression is regulated by a combination of molecular mechanisms; (3) Hi-C spatial interaction intensity is negatively correlated with intergenic distance, and its effect on co-expression dynamically changes with distance, further reflecting the regulatory potential of three-dimensional genome architecture on gene expression. Finally, through integration of co-expression networks and metabolite accumulation data, we identified several key transcription factors potentially involved in metabolic synthesis regulation and found that a subset of SNGs was closely associated with catechin accumulation, especially EGC and EGCG, revealing intrinsic links between SNG co-expression and tissue-specific catechin regulation in tea plants.

In summary, our study advances our understanding of the regulatory mechanisms governing neighboring gene co-expression in tea plants and provides valuable insights and theoretical foundations for future functional genomics research and metabolic regulation related to tea quality traits.

## Data Availability

All data used in this study were obtained from public repositories. The high-quality reference genome of the tea cultivar *C. sinensis* (YK10) was obtained from TeaBase [52]. RNA-seq datasets have been deposited in the Sequence Read Archive (SRA) of the NCBI database under the BioProject number: PRJNA381277, PRJNA524304 and PRJCA009753 [53,54]. ATAC-seq and H3K27ac ChIP-seq datasets were obtained from NGDC under BioProjectPRJCA017759. Hi-C sequencing data were also retrieved from PRJCA017759 [24]. Detailed sample information is provided in Table S3. The genetic data of 11 plant species used for comparative analyses, including gene annotations, CDS sequences, and protein sequences, were downloaded from EnsemblPlants and Phytozome v14.0 [55,56]. Metabolite accumulation data were obtained from previously published studies.

## Supplementary Materials

The following supporting information can be downloaded at: https://www.mdpi.com/article/10.3390/genes17010117/s1, Figure S1: (a) Scatter plot showing the relationship between genomic distance and Pearson correlation coefficient among SNGs. (b) Scatter plot showing the relationship between average gene length and Pearson correlation among SNGs. (c) Co-expression ratio of SNGs under moderate distance and gene length conditions. (d) Co-expression ratio of SNGs with the longest distances and the shortest lengths. Figure S2: Representative example of an enhancer. Figure S3: Scatter plot matrix of molecular features and Pearson correlation coefficients. Figure S4: (a) Co-expression ratio of SNGs sharing different numbers of promoter cis-regulatory elements. (b) Co-expression ratio of SNGs sharing different numbers of GO biological process terms. (c) Co-expression ratio of SNGs across different levels of Hi-C interaction frequency. (d) Co-expression ratio of SNGs under varying expression, ATAC-seq peaks, H3K27ac peaks, and enhancer annotations. Figure S5: (a) Regression coefficients of molecular features from logistic regression models predicting SNG co-expression. (b) Predicted co-expression probability under the interaction between genomic distance and other features. Table S1: Gene ids of specific neighboring gene pairs. Table S2: Genomic locations of enhancers identified in this study. Table S3: Sources of transcriptomic and Epigenomics data used in this study. Table S4: Summary of Predicted Transcription Factors.

## Abbreviations

The following abbreviations are used in this manuscript:

SNGs	specific neighboring gene pairs
GC	gallocatechin
EGCG	epigallocatechin gallate
ECG	epicatechin gallate
EGC	epigallocatechin
EC	epicatechin
C	catechin
KME	key module eigengene-based connectivity
