# Supplementary figures and images for: Multi-Omics Analysis of the Co-Expression Features of Specific Neighboring Gene Pairs Suggests an Association with Catechin Regulation in Camellia sinensis
Source: Genes (Basel). 2026 Jan 22;17(1):117. doi: 10.3390/genes17010117 (PMC12840829; doi:10.3390/genes17010117)

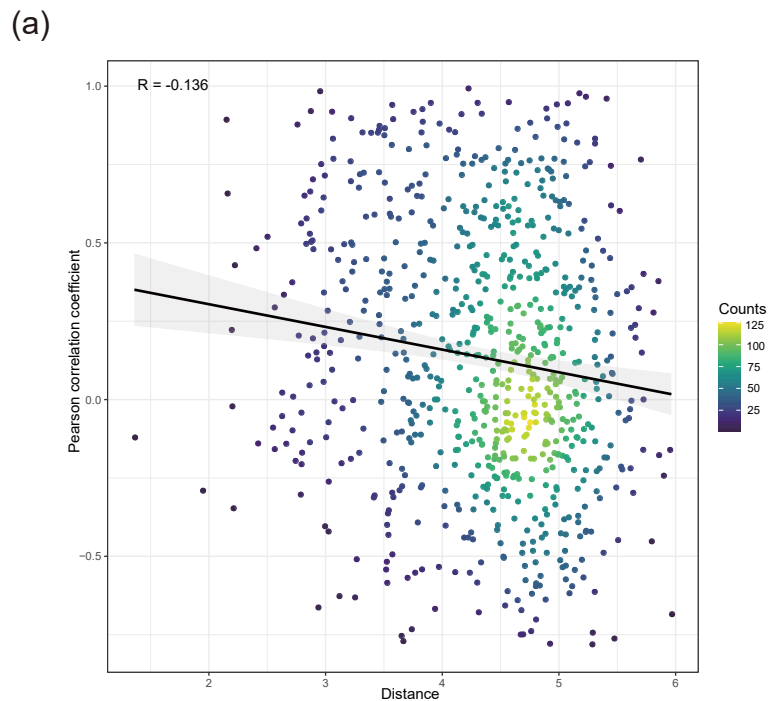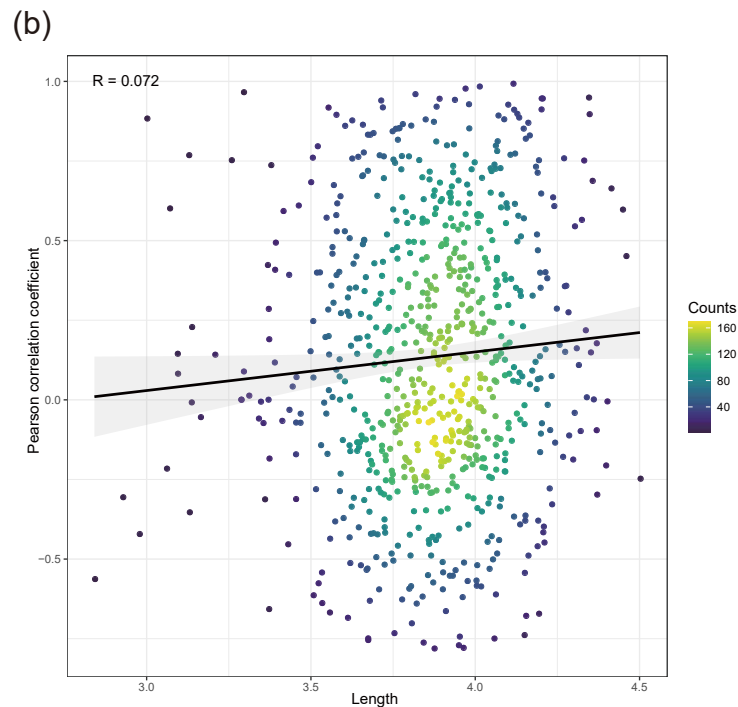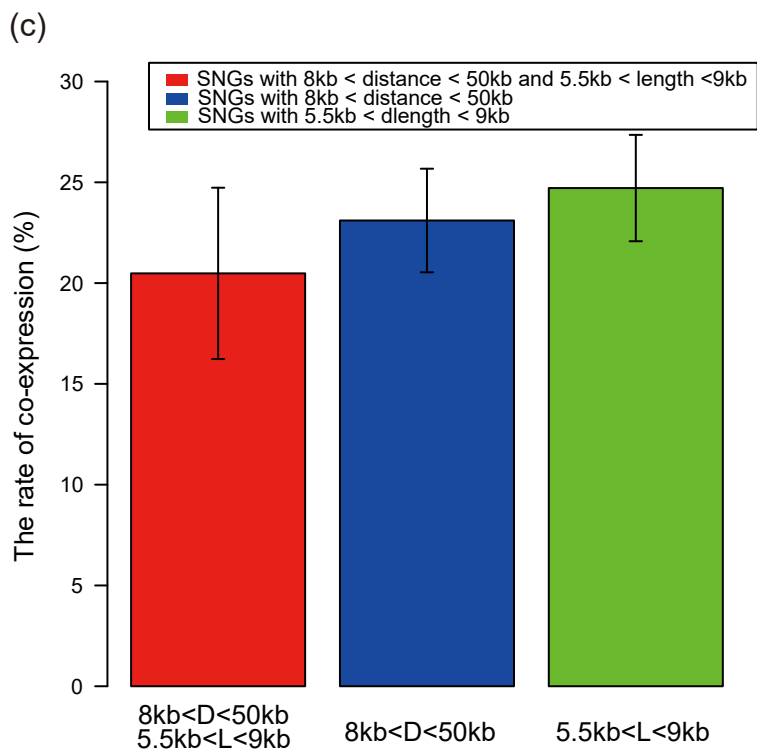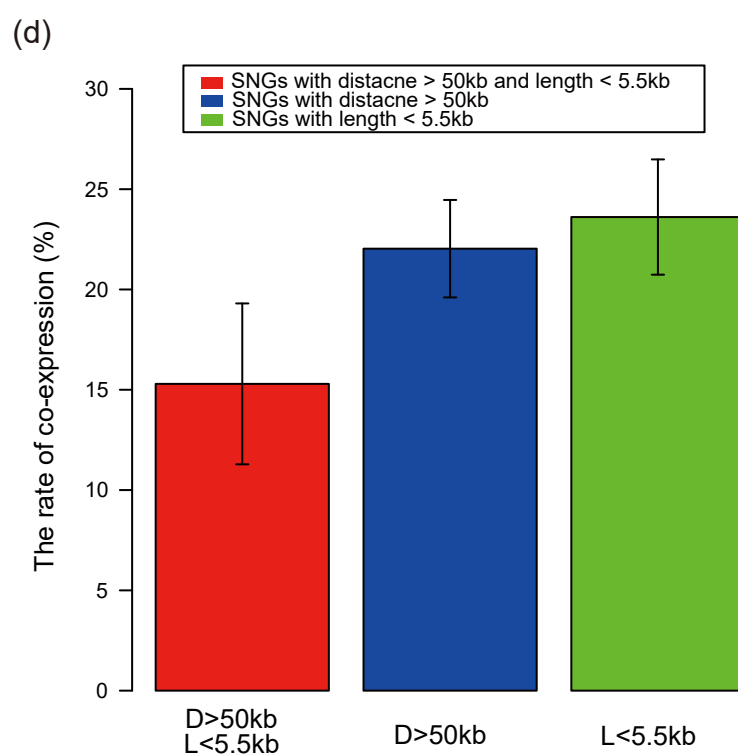

Supplement: Supplementary file 1 [file genes-17-00117-s001.zip › Figure S1.pdf]

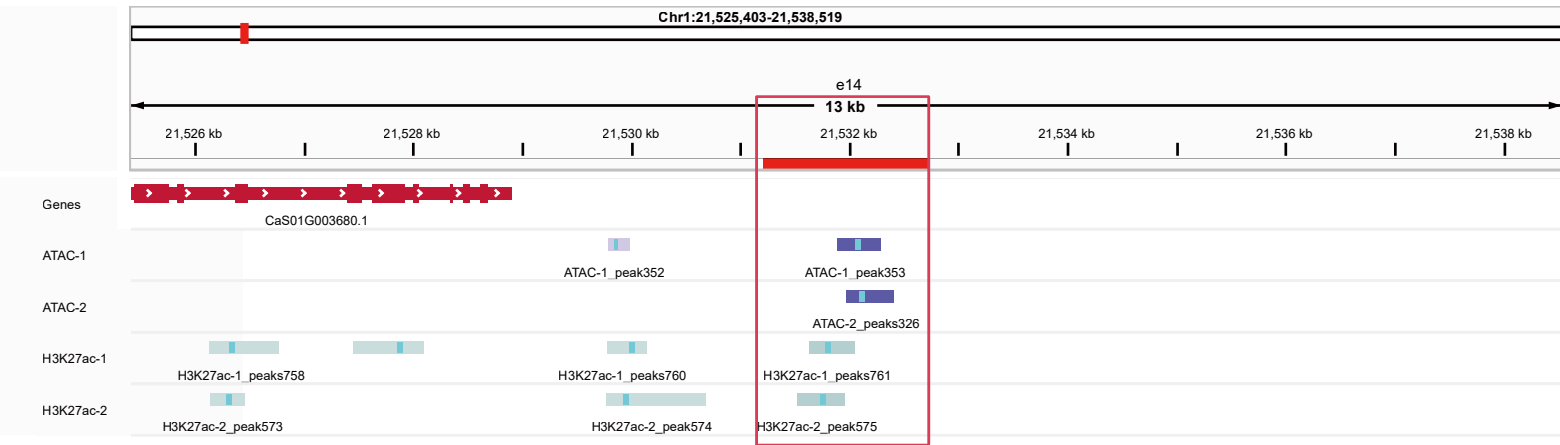

Supplement: Supplementary file 1 [file genes-17-00117-s001.zip › Figure S2.pdf]

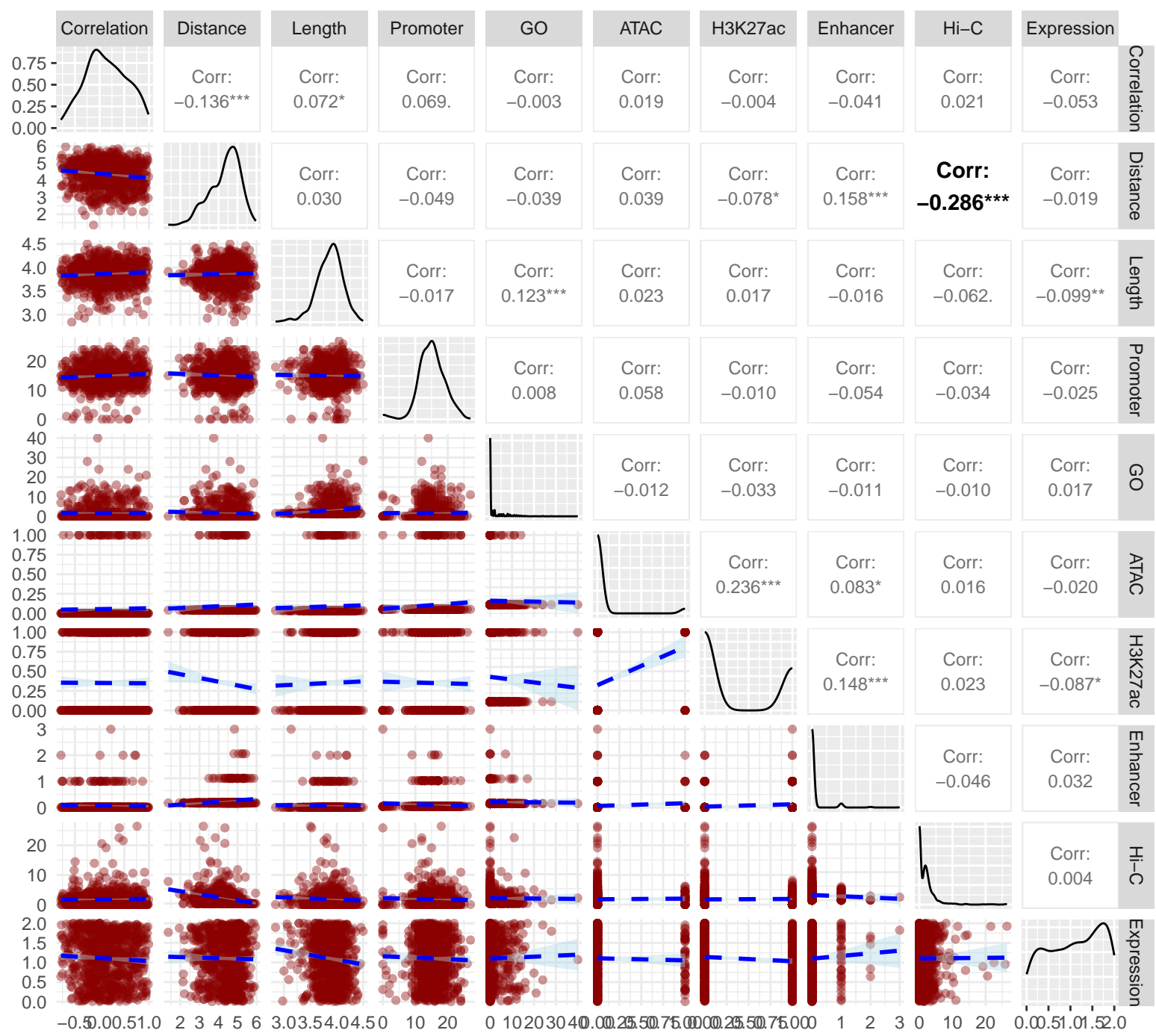

Supplement: Supplementary file 1 [file genes-17-00117-s001.zip › Figure S3.pdf]

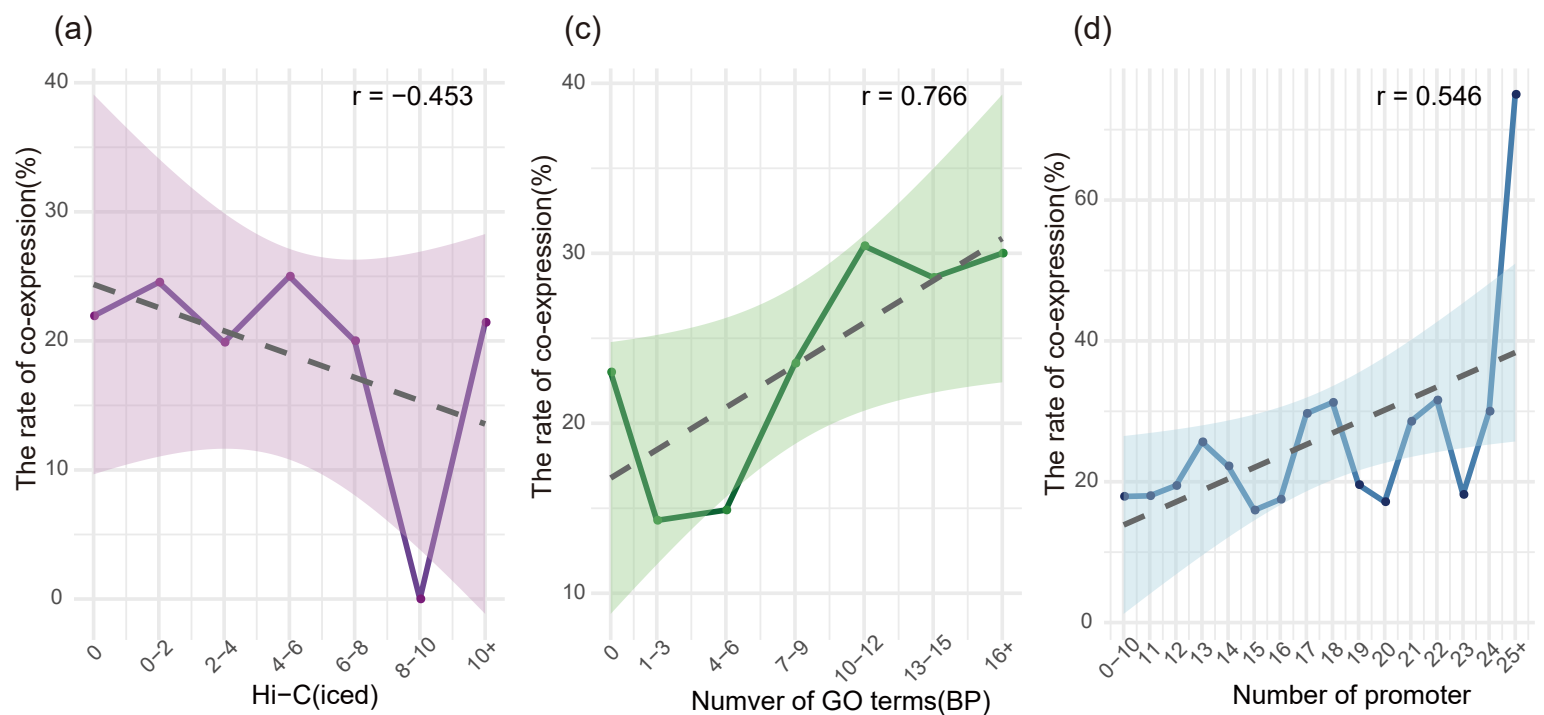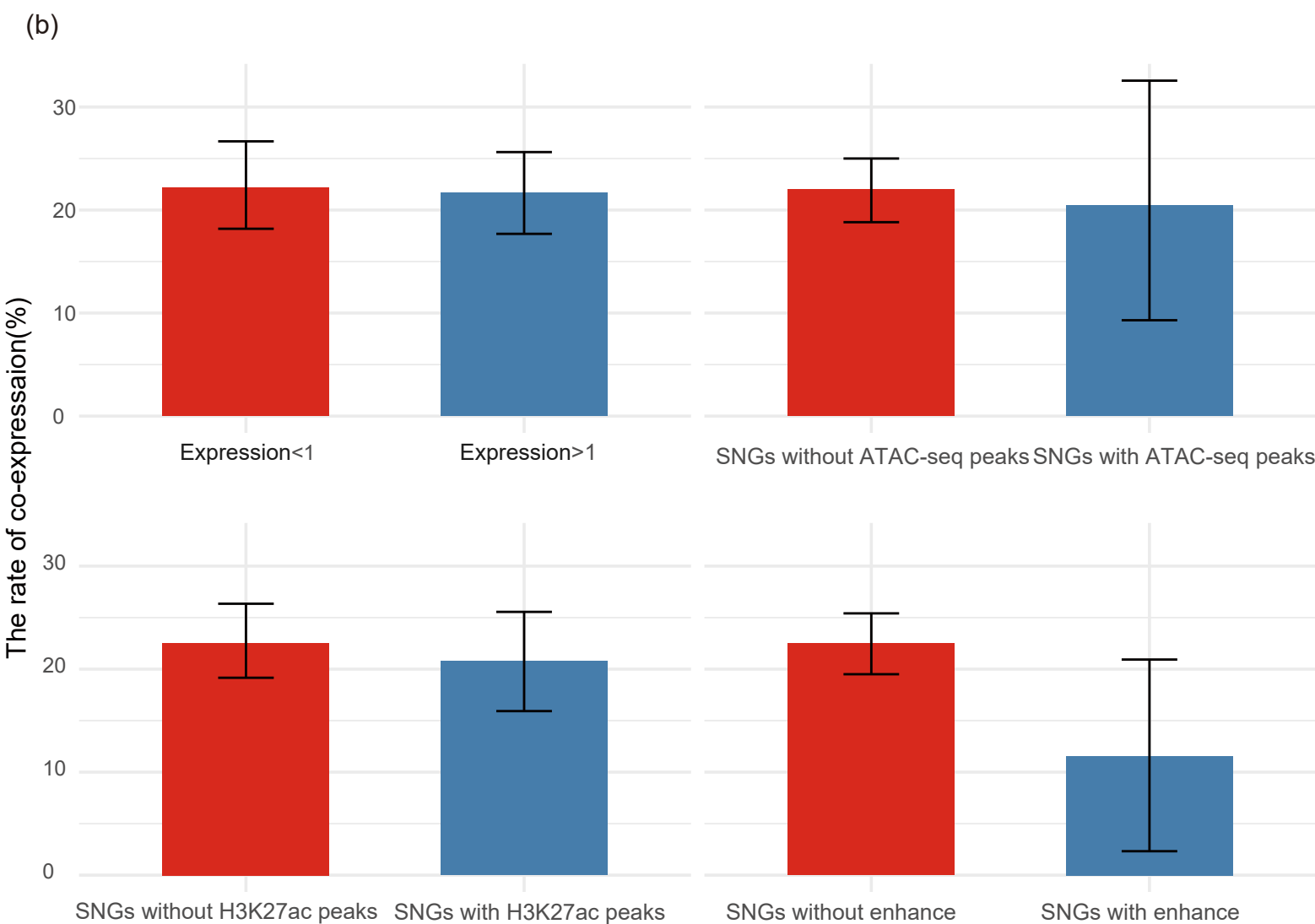

Supplement: Supplementary file 1 [file genes-17-00117-s001.zip › Figure S4.pdf]

(a)

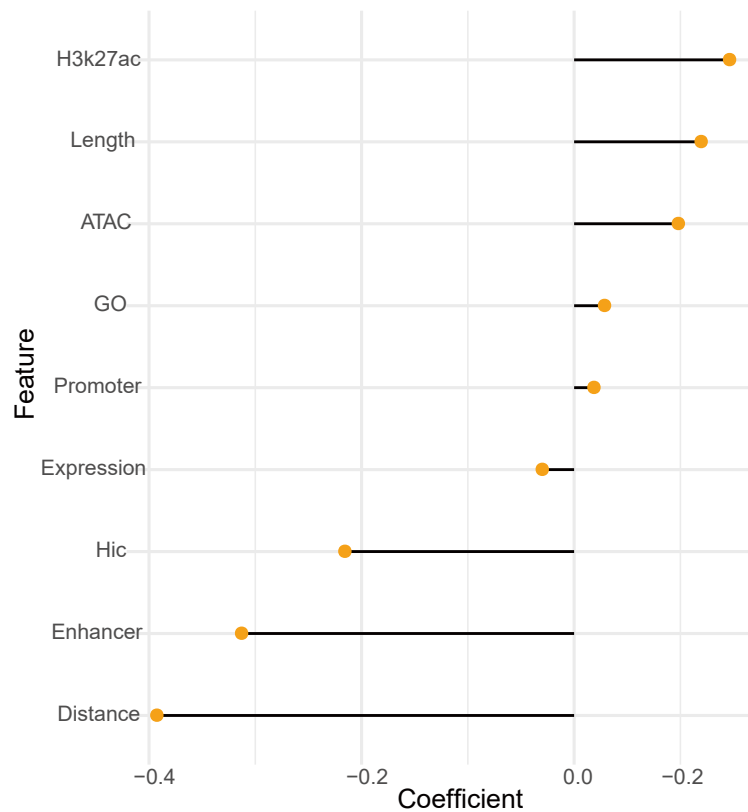

(b)

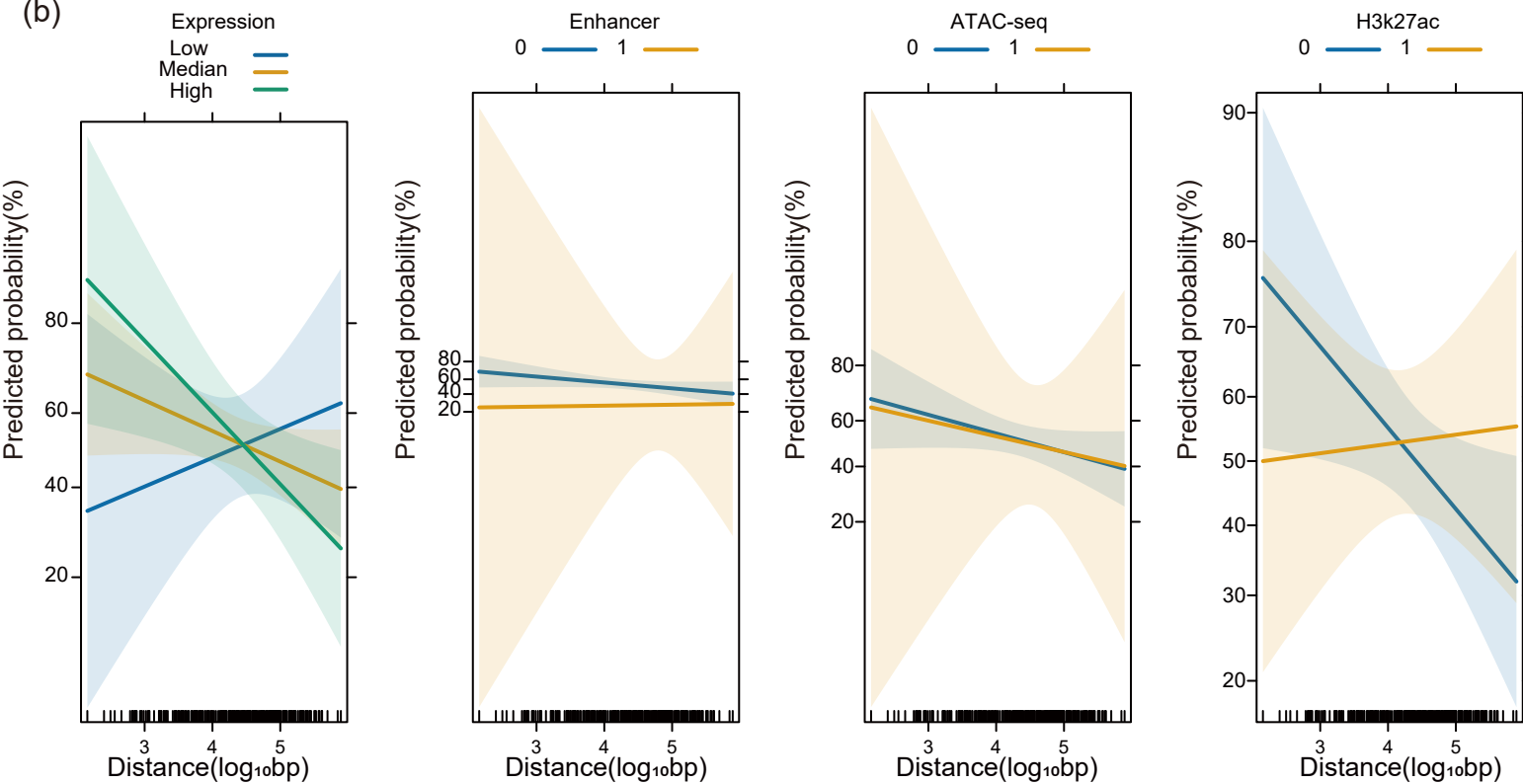

Supplement: Supplementary file 1 [file genes-17-00117-s001.zip › Figure S5.pdf]
